# Stackelberg Game of Buyback Policy in Supply Chain with a Risk-Averse Retailer and a Risk-Averse Supplier Based on CVaR

**DOI:** 10.1371/journal.pone.0104576

**Published:** 2014-09-23

**Authors:** Yanju Zhou, Qian Chen, Xiaohong Chen, Zongrun Wang

**Affiliations:** Business School, Central South University, Changsha, Hunan, China; Middlesex University London, United Kingdom

## Abstract

This paper considers a decentralized supply chain in which a single supplier sells a perishable product to a single retailer facing uncertain demand. We assume that the supplier and the retailer are both risk averse and utilize Conditional Value at Risk (CVaR), a risk measure method which is popularized in financial risk management, to estimate their risk attitude. We establish a buyback policy model based on Stackelberg game theory under considering supply chain members' risk preference and get the expressions of the supplier's optimal repurchase price and the retailer's optimal order quantity which are compared with those under risk neutral case. Finally, a numerical example is applied to simulate that model and prove related conclusions.

## Introduction

In the demand uncertain setting, buyback policy is often adopted by the supplier to encourage the retailer to order more products. The core of buyback policy design is how to formulate proper buyback price to realize win-win between supplier and retailer. Pasternack is the first one that analyzed buyback policy to reach supply chain coordination on the basis of the classical newsvendor problem in 1985 [1]. He classified and discussed contracts according to allowable return quantity (whole or part). From then on, massive related literatures arose, which mainly summarize as the following three aspects: first is to focus on different demand situations, including random demand [2], [3], [5], uncertainty demand [4], and fuzzy demand [6] and so on; second is to discuss the information degree that supplier and retailer are possessed of, including information symmetry or asymmetry [2], [5], [6], [13]–[14]; the third is to explore the risk attitude of decision makers. Here, we mainly pay attention to the third part and its literatures can be classified according to different supply chain network structure and risk attitude hypothesis. Such as, a risk neutral supplier and a risk-averse retailer [7]; a risk neutral manufacturer and two risk-averse retailers [9], [11], [18]; a manufacturer and a retailer are both risk-averse [8], [10] and so on, are investigated respectively. Moreover, Literature [15] led the assumption of risk preference to the research framework of buyback policy and discussed the case of a risk-averse supplier and a risk preference retailer, as well as the case of a risk preference supplier and a risk-averse retailer. Besides, some literatures considered the situation of risk portfolio and risk sharing, e.g. [16], [17] and [19]. The above scholars made important contribution to the research of supply chain coordination.

The motivation of this paper is based on the work of Choi, Li and Yan [8], [10], and they applied the Markowitz's Mean-Variance (MV) model to measure supplier and retailer's risk attitude. Whereas, MV model exists two main inevitable defects, one is that the equal treatment to positive deviation and negative deviation is against the true meaning of risk, and the other is that the probability of profit rate (or “profit” referred in [8] and [10]) must follows a normal distribution. Consequently, this paper adopts Conditional Value at Risk (CVaR) which is widely used in financial engineering (see references in [20]–[21]) to discuss the problem mentioned in [8], that is, how to design buyback policy to realize supply chain coordination in a simple supply chain which is composed of a risk-averse supplier and a risk-averse retailer. Although there are some scholars who adopted CVaR in the research framework of buyback policy [22] and ordering policy [23], our problem is different from theirs. In a word, our work is not simple repeat of the previous studies.

The article differs from previous studies in two aspects. First, we adopt CVaR to measure the retailer's and the supplier's risk attitude, and analyze the buyback policy model under CVaR framework. Second, we have proved that the supply chain coordination can be reached with a risk-averse retailer and a risk-averse supplier, and we get the optimal buyback price of the supplier and the optimal order quantity of the retailer. Moreover, we compare the solution with that under risk neutrality. Hence, our analytical and numerical results lend insights into how a risk-averse supplier designs buyback policy considering that a retailer is also risk averse.

This paper is organized as follows. In section 2, we briefly describe the problem and basic model that will be discussed in the following sections. In section 3, we analyze our single-period buyback policy model based on CVaR. In section 4, we apply a numerical example to simulate the model and prove related conclusions. Finally, in section 5, we draw our conclusions and identify opportunities for future research.

## Problem Description and Basic Model

### 1. Problem description

Consider a bilateral monopoly supply chain. And the supplier provides a single-period short life circle product with unit cost 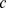 and sells it to the retailer at the wholesale price 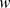, and then the retailer sells it to the consumer at retail price 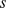. Moreover, in order to encourage the retailer to order more, the supplier will repurchase the unsold products at buyback price 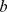 from the retailer. In addition, we give the following assumptions:

1. product demand is stochastic;
2. the information between the supplier and the retailer is symmetrical, that is, the profit function and product demand distribution are known to each other;
3. the supplier and the retailer are both risk-averse, and their targets are to minimize risk at a given certain confidence level;
4. the salvage of unsold product is zero to the retailer, but 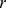 to the supplier;

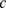
, 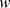, and 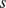 are known or exogenous given.

In order to avoid trivial cases, we assume 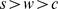, 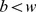 (if 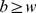, the retailer will order infinite amount of the product because there is always a profit margin out of it), and 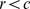 (if 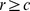, the supplier will produce as many products as possible).

The decision variables are buyback price 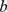 and order quantity 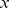. In general, the buyback policy decision-making can be regarded as a process of Stackelberg game. Before selling season, the supplier provides wholesale price and buyback price to the retailer, and then the retailer determines the optimal order quantity. Based on the information symmetry hypothesis, the supplier knows the ordering choice of the retailer; therefore he will decide the optimal buyback price accordingly.

### 2. Basic model

#### Profit function

The retailer's profit function can be given as
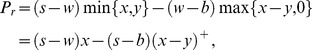
(1)where 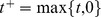, 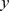 expresses product market demand, 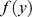 and 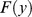 are demand density distribution function respectively.

The supplier's profit function is 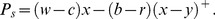
(2)


The supply chain's profit function is 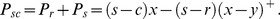
(3)


#### CVaR function

The meaning of CVaR originated from VaR (Value at Risk). VaR means the possible maximal loss which some properties or property portfolio could suffer in a specific time at the given confidence level under the normal market condition. As VaR can't involve the situation when the loss goes beyond the maximum possible loss at the given confidence level, namely ignores tail risk, CVaR is introduced. CVaR means the conditional expectation when loss exceeds VaR. Whatever loss distribution follows, CVaR has the properties of positively homogeneity, sub-additive, monotonicity and translational invariance, thus it is coherent. Let 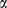 be VaR, and 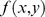 be the loss function. Here 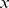 is the decision variable and 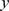 is the random variable (which are the same as the above). 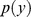 is the density function. CVaR, denoted by 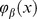, is 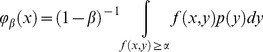
(4)


Rockafellar and Uryase [12] had proved that we can define a much simpler function which can be used instead of CVaR, i.e.
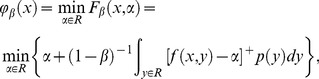
(5)where 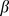 is the degree of confidence, and 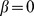 means risk neutral. Furthermore, it can be proved: (1) 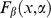 is convex with respect to 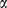; (2) VaR is the minimum value of 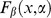 with respect to 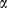.

Denote 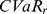 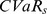, and 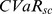 be CVaR functions of the retailer, the supplier, and the supply chain respectively. Hence, we have
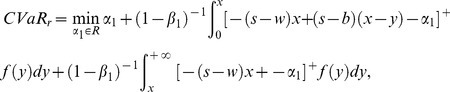
(6)

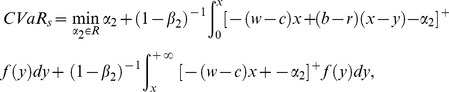
(7)where 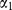 and 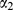 represent VaR of the retailer and the supplier respectively, and 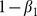 and 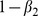 represent CVaR's confidence level of the retailer and the supplier respectively.

Suppose that supply chain's risk consists with the retailer's and the supplier's risk, and thus we adopt additive model to calculate supply chain's risk. Obviously that is consistent with the reality, and therefore we have



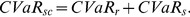
(8)


### 3. Buyback policy model

#### Analysis of the retailer's CVaR

Three CVaR expressions of the retailer are obtained according to the value range of VaR.

CASE I. If 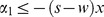, then 
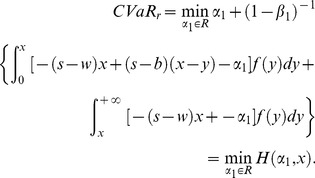
(9)


After taking the first partial derivatives of (9) with respect to 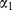, we have
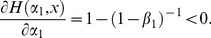
(10)


Evidently, 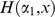 is the decreasing function with respect to 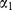 Therefore, when 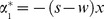, 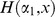 takes minimum value, and we can rewrite (9) as follows:
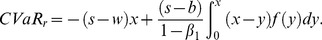
(11)


CASE II. If 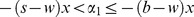, then 
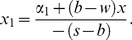
(12)


It can be easily proved that 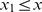, and then we can get
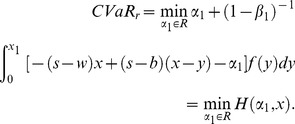
(13)


In a similar way to the above, we get the first partial derivative of (13), namely the first order condition is
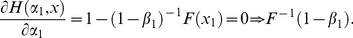
(14)


Obviously, 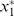 satisfies (12), and then we have
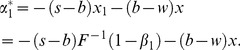
(15)


Therefore, (13) can be defined as
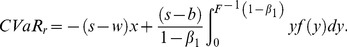
(16)


CASE III. If 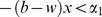, then 
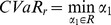
. It is obvious that we can get CVaR value when 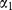 is minimum. Here the optimization problem of CVaR function is similar to CASE II.

Consequently the retailer's buyback policy can be summarized as the following two settings according to the region of order quantity:

1. if 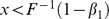, then 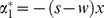, and the retailer's 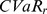 can be expressed by (11);
2. if 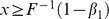, then 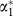 is (15), and the retailer's 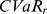 can be expressed by (16).

#### Analysis of the supplier's CVaR function

The analysis of the supplier's CVaR function is similar to the above. Therefore we omit it and just show the final results according to the retailer's order quantity region,

1. If 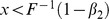, then 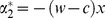. The supplier's CVaR function can be given as:

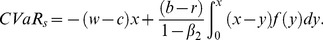
(17)2) If 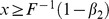, then 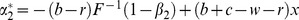. And the supplier's CVaR function is as follow
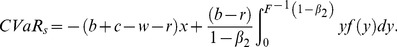
(18)

#### Analysis of supply chain's CVaR function

According to the supply chain's CVaR function, namely (8), we discuss the relation between 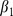 and 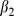, and then determine its expression.

If 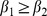, obviously we can get 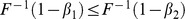, because 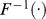 is a strictly increasing function. Thus we can discuss the expression of 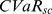 according the following situation.

1. If 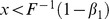, then the supply chain's CVaR, 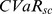, consists of (11) and (17) and it can be derived as follows:

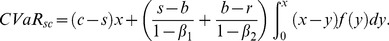
(19)2) If 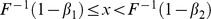, then 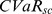 consists of (16) and (17), namely

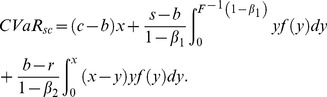
(20)3) If 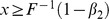, then 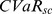 consists of (16) and (18), that is

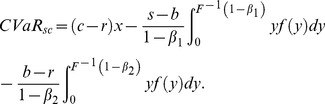
(21)In contrast, if 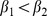, then 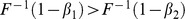. The calculation of the supply chain's CVaR is just as the above derivation process and we have:

1. If 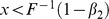, then 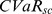 is the same as (19);
2. If 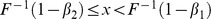, then (18) plus (11) gives the following,

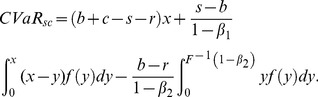
(22)3) If 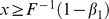, then 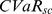 is the same as (21).

In conclusion, the above analysis obtains all expressions of 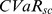 via the discussion of relation between 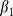 and 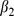. Thus we can begin to discuss the decision-making process of the supplier and the retailer by means of minimizing 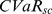.

#### Stackelberg game of buyback policy based on CVaR

In the first stage of Stackelberg game, the retailer determines optimal order quantity with the target of minimizing risk value at the given buyback price and wholesale price. Therefore, we get the optimal order quantity expression of the retailer firstly. Since we have got the CVaR expression of retailer, the first derivatives with respect to order quantity of (11) and (16) can be given as follows:
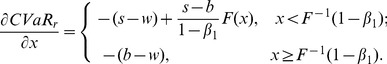
(23)


Set (23) be zero, namely the first order condition equals to zero, and then we can obtain the optimal order quantity 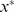. Since 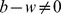, the optimal order quantity is calculated by
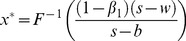
(24)satisfying 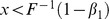. Consequently it is the feasible solution of the retailer's optimal order quantity.

Similarly, we can get the first derivative of the supply chain's CVaR with respect to order quantity of (19), (20), (21) and (22).

1. If 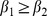, then

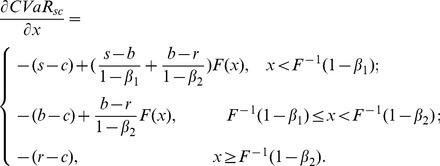
(25)Set (25) be zero and then we obtain the supply chain's optimal order quantity 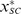. Obviously, when 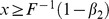, there is no feasible solution for 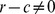. When 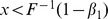, the optimal order quantity,
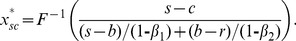
(26)lies in the region, and therefore it is feasible. However, when 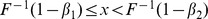, the solution doesn't satisfy (25), namely it doesn't fall in the range of 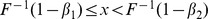. So we ignore it.

1. If 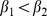, then

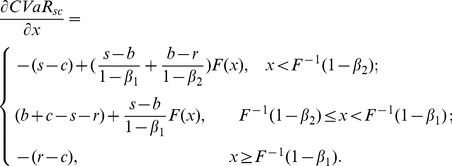
(27)Similarly, we can obtain the supply chain's optimal order quantity 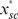 under the condition of 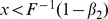, and it is easily proved that this is a feasible solution. Thus we express it as follows, 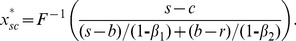
(28)


Obviously, (28) is the same as (26). In addition, the other solution 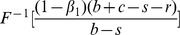 isn't in the region of 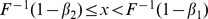, so we ignore it.

From the above analysis, the supply chain's optimal order quantity with the target of minimizing CVaR can be expressed as (26) or (28) in spite of the relation between 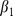 and 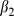.

It is well known that if the supplier and the retailer are both risk neutral and maximizes their own profits, double marginalization will prevail. That is, the retailer's optimal order quantity is less than the integrated supply chain's. Additionally, reference [1] had also proved that the retailer's optimal order quantity is lower than supply chain's optimal order quantity when the supplier doesn't adopt buyback policy. When the supplier adopts buyback policy, that is, a part of demand risk is untaken by the supplier, the retailer will be urged to improve order quantity to reach supply chain's optimal order quantity. Then we can obtain the optimal buyback price expression of the supplier by making the retailer's optimal order quantity equal to the supply chain's. Thus it reaches supply chain's coordination.

As a result, in the second stage of Stacklberg game, set 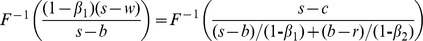. Therefore, the supplier's optimal buyback price satisfies the following equation,
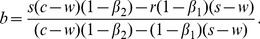
(29)


The supplier encourages the retailer's order quantity by buyback policy, and thus it reduces double marginalization effect and achieves supply chain coordination. Especially, when 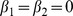, it illustrates that the retailer and the supplier are both risk neutral, and CVaR minimization equals to expected profit maximization. Therefore, the retailer's optimal order quantity is 
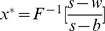
, and the supply chain's optimal order quantity is 
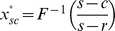
. Thus the supplier's optimal buyback price satisfies 
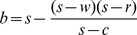
. From the above analysis, we can get the following conclusions.

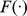
 is a strictly increasing function. Therefore 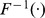 is also an increasing function. Obviously, we have 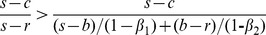, 
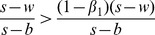
, and thus we can get that the supply chain's optimal order quantity in risk neutrality is more than that in risk aversion. In another word, the optimal order quantity decreases as risk aversion degree of the retailer and the supplier increase.
2. The expressions of the retailer's 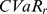 are (11) and (16). If 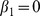, 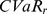 reaches minimum, and decreases as 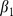 increases. Consequently, the retailer's CVaR in risk neutral is higher than that in risk-averse, and increases as the retailer's risk aversion degree increase. Furthermore, the increase in CVaR equals to negative profit's increase, namely profit's decrease. The regularity of the supplier's CVaR is similar to that.
3. If 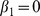, then the expression of the supplier's optimal buyback price yields the following form 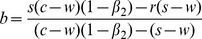, and obviously it decreases as 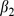 increases (*Property1*). Therefore we can conclude that the supplier's buyback price decreases as the supplier's risk aversion degree increases when the retailer is risk neutral. That is to say the risk which the risk-averse supplier is willing to share is less than the risk–neutral supplier does.
4. If 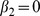, then the supplier's optimal buyback price can be expressed by 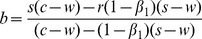, and it increases as 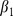 increases (*Property2*). Similarly we can conclude that the supplier's buyback price rises as the retailer's risk aversion degree rises. Namely, in order to encourage the retailer's order more, the supplier needs to endure more risk when the retailer is risk averse than that when the retailer is risk neutrality.

***Property1.*** If 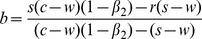, then 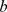 is decreasing in 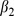.

Proof.

After taking the first derivatives of 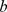 with respect to 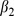, we get
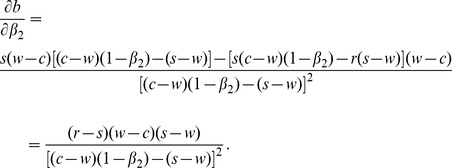
(30)


Because 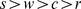, expression 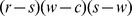 is negative. Thus we have 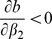.

***Property2.*** If 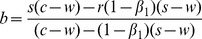, then 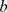 is increasing in 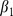.

Proof.

After taking the first derivatives of 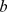 with respect to 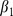, we get
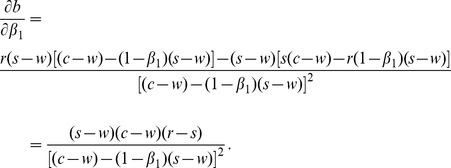
(31)


Because 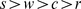, expression 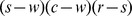 is positive. Thus we have 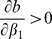.

## Numerical Calculation

Suppose product demand follows normal distribution, namely 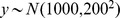, 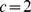, 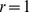, 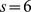, and 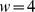. According to the previous analysis, 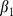 and 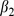 represent risk aversion degree of the retailer and the supplier respectively. The more 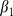 and 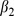 are, the higher risk aversion degree of the retailer and the supplier are. Thus we will obtain the optimal buyback price by different value of 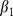 and 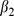 in light of (29), and the results are illustrated in table 1.

From the table 1, we can conclude that the buyback prices are the same when the risk aversion degree of the supplier equals to the retailer's. And when 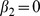 and 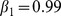, the supplier's optimal buyback price is the highest, i.e., 5.9504. In other words, the supplier's buyback price and taking risk as the risk aversion degree of the retailer increases. Furthermore, when 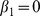 and 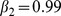, the buyback price is lowest. In brief, when the supplier's risk aversion degree is higher, he only pays the lower buyback price, that is, he is unwilling to undertake more risk.

Table 2 shows the supply chain's optimal order quantity in different 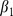 and 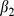.

**Table 1:** The supplier's optimal buyback price.

	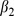					
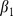	0	0.8	0.85	0.90	0.95	0.99
0	3.500	1.8333	1.6521	1.4545	1.2380	1.0495
0.8	5.1666	3.5000	3.14285	2.666	2.000	1.238
0.85	5.3478	3.8571	3.500	3.000	2.2500	1.3125
0.90	5.5454	4.3333	4	3.500	2.6666	1.4545
0.95	5.7619	5.000	4.7500	4.3333	3.500	1.8333
0.99	5.9504	5.76196	5.6875	5.5454	5.1662	3.500

**Table 2:** The supply chain's optimal order quantity.

	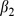					
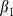	0	0.8	0.85	0.90	0.95	0.99
0	1168	990	980	970	959	951
0.8	990	801	784	765	744	724
0.85	980	784	765	744	719	696
0.90	970	765	744	719	689	659
0.95	959	744	719	689	650	605
0.99	951	724	696	659	605	518

We can see that the optimal order quantity decreases as the risk aversion degree of the supplier and the retailer increases. If 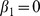 and 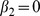, then that means when the supplier and the retailer are both risk neutral, the supply chain's optimal order quantity is the largest; and if 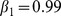 and 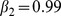, then it is the lowest.

**Figure 1:**
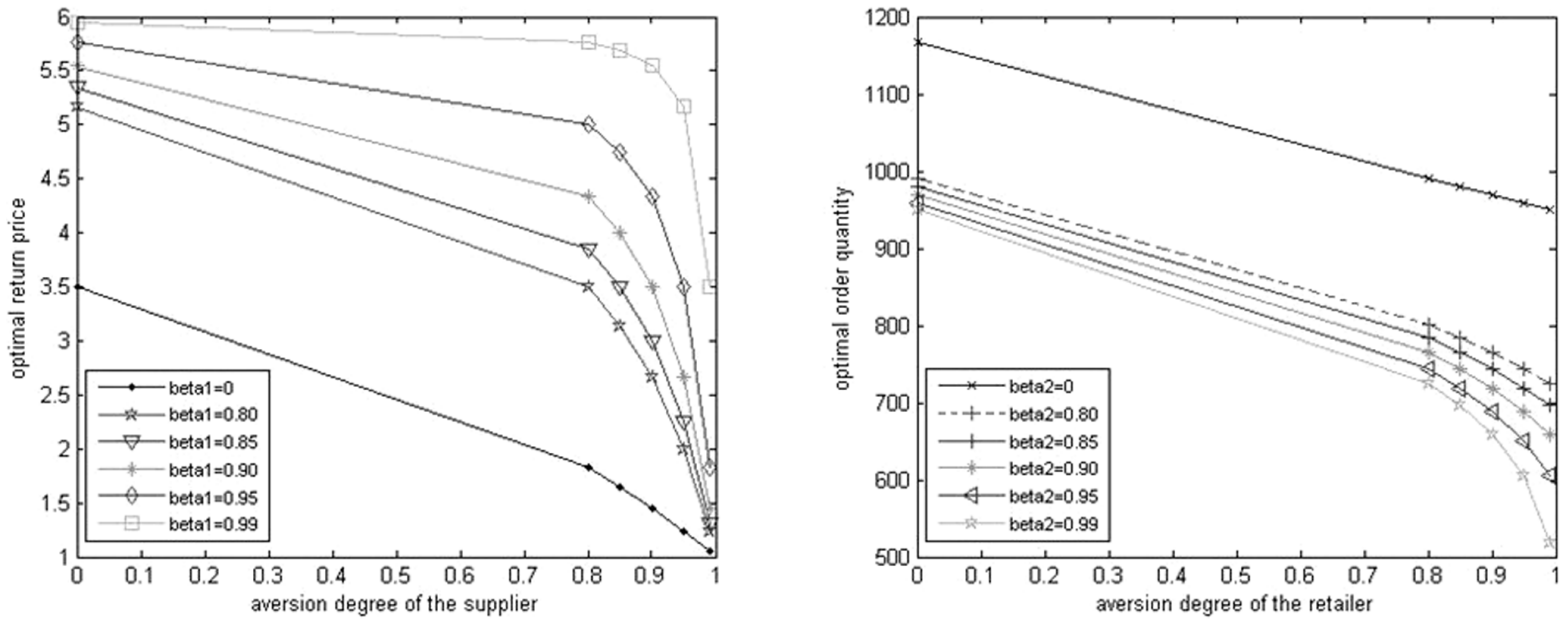
Optimal buyback price and order quantity under different risk aversion degree.

**Figure 2:**
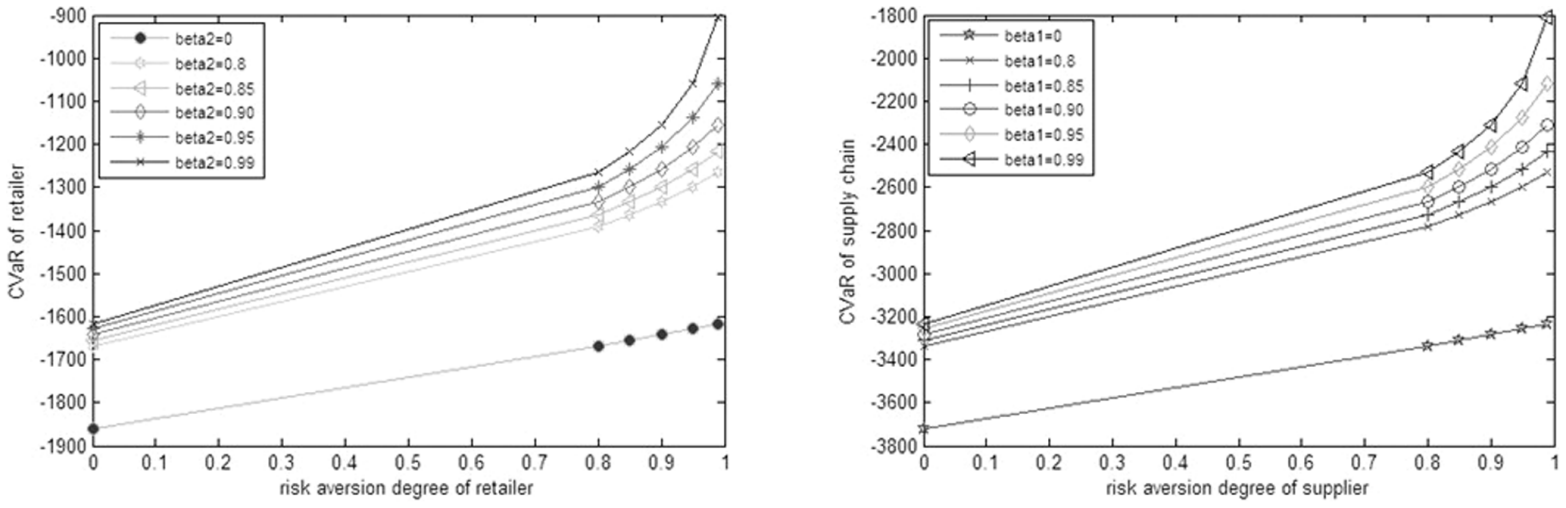
CVaR of retailer and supply chain under different risk aversion degree.

Figure 1 shows the trend of the optimal buyback price and optimal order quantity directly. The left figure shows the optimal buyback price under different risk aversion degree of the retailer and the supplier. And the curve shows the supplier's optimal Stackelberg game buyback price according to its own different risk aversion degree at the given the retailer's risk aversion degree, e.g. beta1 = 0, which represents the bottom curve. Different curve stands for different risk aversion degree of the retailer 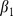. And then it can be easily known that the buyback price and the slope of curve are decreasing as the supplier's risk aversion degree is increasing when the retailer's is certain. That is to say the rate of descend is increasing. From the vertical direction, the supplier's optimal buyback price is increasing as the retailer's risk aversion degree is increasing when the supplier's is certain, and this is consistent with the reality. The right figure is the optimal order quantity under different risk aversion degree of the retailer and the supplier. And every curve represents the retailer's optimal order quantity according to its own different risk aversion degree under fixing the supplier's risk aversion degree 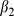. Different curve shows different risk aversion degree of the supplier. From figure 1, we can conclude that when the supplier's aversion degree is certain, the optimal order quantity is decreasing as the retailer's risk aversion degree is increasing. From the vertical direction, the optimal order quantity and the slope of the curve is decreasing as the supplier's risk aversion degree is increasing when the retailer's aversion degree is certain, in other words, the descend rate of the optimal order quantity is increasing. In conclusion, policymaker will reduce risk by reducing optimal quantity whereas the retailer's or the supplier's risk aversion degree increases. In addition, we put the supplier's optimal buyback price and the supply chain's optimal order quantity from the Table 1 and Table 2 into the equations (10), (16) and (18), and thus we can obtain the CVaR values of the retailer, the supplier and the supply chain respectively. For the addition of the first two equals to the latter, we just need to solve the two of them (See figure 2).

Net loss is represented by negative profit, so we can get the trend of profit from the trend of CVaR's absolute value. From the figure 2, we can see that the trend of the retailer's CVaR is the same to the supplier's CVaR, and therefore we just need to analyze the trend of the supply chain's CVaR in the right figure. And it is the supply chain's CVaR under the different supplier's risk aversion degree at the given aversion degree of the retailer, e.g. beta1 = 0.95, and different curve represents different risk aversion degree of the retailer. In horizontal direction, the supply chain's CVaR is increasing as the supplier's risk aversion degree is increasing at the certain risk aversion degree of the retailer, that is to say the supply chain's profit is decreasing. In the vertical direction, the supply chain's CVaR is increasing as the retailer's aversion degree and the slope of curves is increasing at the certain aversion degree of the supplier. Thus the more risk-averse the decision makers are, the faster the CVaR increases. This is equals to that the descend rate of the supply chain's profit is increasing, and obviously it is consistent with reality.

## Conclusions

In this paper, we discuss the coordination of buyback policy in the supply chain composed of a risk-averse retailer and a risk-averse supplier, and adopt CVaR to depict their risk attitude. Then we conduct Stackelberg game to describe the coordination process of buyback policy. Finally, we draw the following conclusions:

1. The retailer's optimal order quantity in risk neutrality is more than that in risk aversion. In other words, the optimal order quantity decreases as the risk aversion degree of the retailer and the supplier increase.
2. The CVaR value of policymakers in risk neutrality is less than that in risk aversion. And CVaR is increasing as the risk aversion degree of the retailer and the supplier is increasing. This equals to the increase of negative profit or the decrease of the profit.
3. When the retailer is risk neutral, the higher the supplier's risk aversion degree is, the lower the supplier's optimal buyback price is, in other words, the risk which the supplier is willing to share decrease as the increase of the supplier's risk aversion degree.
4. When the supplier is risk neutral, the higher the retailer's aversion degree is, the higher the supplier's optimal buyback price is, that is to say the risk which the supplier is willing to share increase as the increase of the retailer's risk aversion degree.

The above-mentioned conclusions based on CVaR can be applied to the channel coordination of buyback policy in the real supply chain setting because, in general, most of the participants are risk-averse when they are facing uncertain demand. The paper provides us the meaningful managerial insights, which show what buyback policy a risk-averse supplier design when he faces a risk-averse retailer. It would be interesting to extend our research to investigate the risk aversion of the supply chain members in the asymmetric situation in which cost information of the retailer is unknown to the supplier.
